# Keeping the Balance Between Proliferation and Differentiation: The Primary Cilium

**DOI:** 10.2174/138920211795860134

**Published:** 2011-06

**Authors:** Florencia Irigoín, Jose L Badano

**Affiliations:** 1 Institut Pasteur de Montevideo, Facultad de Medicina, Universidad de la República, Montevideo, Uruguay; 2 Departamento de Histología y Embriología, Facultad de Medicina, Universidad de la República, Montevideo, Uruguay

**Keywords:** Cilia, cell cycle, ciliopathies, cancer.

## Abstract

Primary cilia are post-mitotic cellular organelles that are present in the vast majority of cell types in the human body. An extensive body of data gathered in recent years is demonstrating a crucial role for this organelle in a number of cellular processes that include mechano and chemo-sensation as well as the transduction of signaling cascades critical for the development and maintenance of different tissues and organs. Consequently, cilia are currently viewed as cellular antennae playing a critical role at the interphase between cells and their environment, integrating a range of stimuli to modulate cell fate decisions including cell proliferation, migration and differentiation. Importantly, this regulatory role is not just a consequence of their participation in signal transduction but is also the outcome of both the tight synchronization/regulation of ciliogenesis with the cell cycle and the role of individual ciliary proteins in cilia-dependent and independent processes. Here we review the role of primary cilia in the regulation of cell proliferation and differentiation and illustrate how this knowledge has provided insight to understand the phenotypic consequences of ciliary dysfunction.

## 1. INTRODUCTION

**Fig. (1) The basic structure of cilia.**
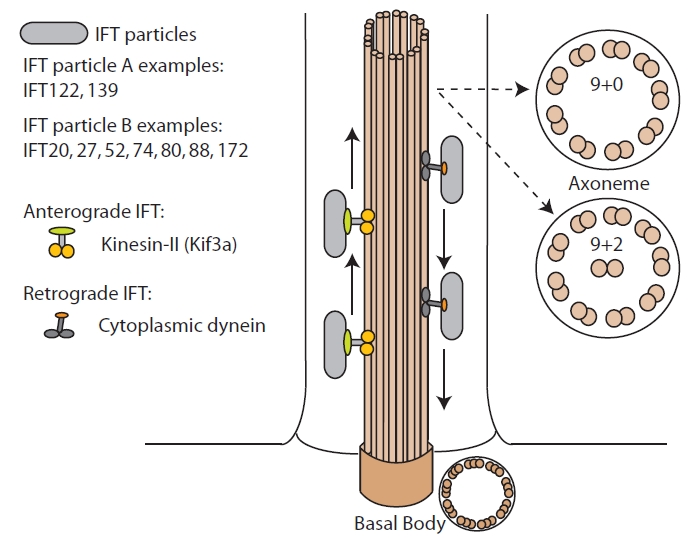
Schematic representation of the cilium showing the organization of the microtubule backbone (axoneme and basal body) and the process of IFT. The two main types of axoneme, 9+2 and 9+0 are shown and some examples of IFT particles are highlighted.

The regulation of cell fate decisions such as whether to divide or differentiate is a key aspect of development as well as to maintain the homeostasis of tissues and organs in the adult organism. A complex array of pathways and signaling cascades has been implicated in these processes and more recently, a large body of data has highlighted the central role of a particular organelle, the primary cilium. Primary cilia are antennae-like cellular protrusions that are present in almost every cell type in the human body (http://www. bowserlab.org/primarycilia/ciliumpage2.htm). These organelles consist of a microtubule based backbone, the axoneme, composed of nine microtubule doublets that are organized from a basal body, a structure derived from the mother centriole of the centrosome that is composed of nine microtubule triplets [1]. Unlike motile cilia and flagella where the nine axonemal microtubule doublets surround a central pair (9+2 configuration), primary cilia typically present a 9+0 axonemal configuration and are generally non motile structures, albeit exceptions can be found (Fig. **1**) [1, 2]. For the formation, maintenance and function of cilia, a specialized transport mechanism termed intraflagellar transport (IFT) uses the molecular motor activity of kinesin-II and cytoplasmic dynein 2 to transport IFT cargo in and out of the cilium respectively (Fig. **1**) [3, 4].

Primary cilia have been shown to represent key structures in the integration and transduction of a range of stimuli to coordinate and regulate cell fate. Consequently, it is not surprising that ciliary dysfunction plays a major role in the etiology of several human conditions that have been grouped under the name of ciliopathies to denote their common or shared cellular basis (for some extensive reviews on the topic see Refs. [2, 5-8]). Importantly, the ciliopathies share, to a variable extent, a set of what have been recognized as cilia associated phenotypes that include cystic kidney disease, obesity, malformations of the central nervous system, retinal degeneration and asthma among other clinical manifestations (Table **1**). 

The broad phenotypic consequences of ciliary dysfunction are due to a number of factors including the nearly ubiquitous presence of cilia in the human body and the fact that these organelles participate in a number of important biological processes. To date we know that primary cilia can act both as mechano and chemo-sensors that for example modulate intracellular calcium levels or help establish the left-right body axis [2, 5, 6, 9]. In addition, these organelles actively participate in the transduction of different signaling cascades including Wnt, Hedgehog (Hh) and platelet derived growth factor (PDGF) [10-14]. Moreover, several ciliary proteins have been shown to have important extra-ciliary functions thus complicating the dissection of truly cilia-associated phenomena. Here we review the function of primary cilia in cell fate decisions focusing on the role of this organelle in keeping the balance between proliferation and differentiation, an aspect clearly affected upon ciliary malfunction and a hallmark of different cilia-associated phenotypes. We therefore provide examples of the multiple ways by which this complex organelle can influence cellular and tissue homeostasis to finally briefly mention how this knowledge has provided insight to understand the cellular basis of the ciliopathies. 

## 2. CILIA BIOGENESIS AND THE CELL CYCLE: THE CILIARY CYCLE

**Fig. (2). The cell and ciliary cycle.**
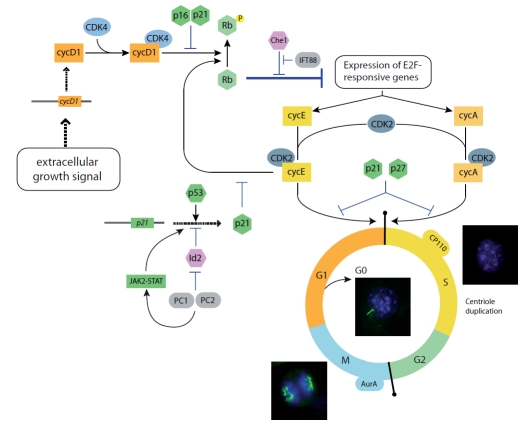
Simplified representation of the control of the cell cycle where we have incorporated ciliary proteins that have been described as directly implicated in cell cycle control. Immunocytochemistry images correspond to NIH3T3 fibroblasts in different stages of the cell cycle, stained with anti-acetylated tubulin (green) to visualize the primary cilium, anti-γ tubulin (green) for staining the centrosome and DAPI for the nucleus. Cyc: cyclin; CDK: cyclin dependent kinase.

Cilia are organized from a basal body that derives from the mother centriole of the centrosome which migrates and docks in close proximity to the apical plasma membrane of the cell. Upon cytokinesis, each daughter cell inherits one centrosome that will become the microtubule organizing center [15]. As cells engage in the cell cycle, centrosomes are duplicated during the G1/S transition [16] and separate during G2 to become the poles of the mitotic spindle. Thus, the organization of cilia from a basal body and the life cycle of centrosomes and cell division are tightly regulated and linked to each other (Fig. **2**) (for in depth reviews on the topic see Refs. [17, 18]). 

Primary cilia are post-mitotic cellular structures that are present in cells during G0/G1 and the beginning of S phase and disassemble at late S phase or beginning of G2 when centrioles, including the one functioning as a basal body, are needed to organize the mitotic spindle (Fig. **2**) [19]. However, the link between the ciliary and the cell cycles not only relies in the availability of centrioles. This is supported by the fact that in some cell types the cell cycle can proceed even in the absence of centrioles, albeit with a reduced efficiency and suffering of disorganized mitotic spindles (for some references see [20-23]; reviewed in detail in Ref. [15]). In contrast, several proteins involved in cell division actively participate in the control of ciliogenesis and vice versa, some ciliary proteins have been claimed to directly regulate the cell cycle (discussed in the next section). 

One example of proteins associated with cell division, in particular centrosome duplication and cytokinesis, that appears to participate in the ciliary cycle is CP110 [24]. It has been shown that CP110 interacts with Cep97 and CEP290, a protein that has been causally associated with several ciliopathies, to inhibit ciliogenesis [2, 25, 26]. Spektor and colleagues showed that Cep97 targets CP110 to the centrosome. Both proteins appear to be necessary for inhibiting ciliogenesis as depletion of either of them promotes ciliary assembly in proliferating cells. Conversely, overexpression of CP110 in non dividing cells inhibits cilia formation [25]. Subsequently, it was shown that the inhibitory effect of CP110 over ciliogenesis is mediated by its interaction with CEP290 in discrete protein complexes where CP110 antagonizes the ciliogenic effect of CEP290 [26]. Importantly, the expression pattern of CP110 along the cell cycle is opposite to the ciliation status, consistent with its inhibitory effect on ciliogenesis: it is highly expressed during the G1/S transition and is almost undetectable during G0 [24]. Interestingly, in quiescent cells CP110 has been shown to be specifically depleted only from the mother centriole, which will become the basal body, while it continues to be present in the daughter centriole [25]. Thus, regulating proteins with opposite roles in the context of the centrosome and the cilium might represent an efficient mechanism to coordinate ciliogenesis with cell proliferation. This effect could be achieved not only by modulating the amounts of individual proteins but also by controlling their participation in defined, separable protein complexes. 

As previously mentioned, primary cilia are inherently post-mitotic structures and thus ciliary disassembly also needs to be coordinated with the entry into mitosis. Aurora A (AurA) is a centrosomal protein involved in the regulation of mitotic entry and that have been shown to participate in the disassembly of primary cilia. Upon mitotic stimulation *via *growth factors, the focal adhesion protein HEF1 phosphorylates and activates AurA at the basal body of cilia. In turn, activated AurA phosphorylates and activates the histone deacetylase HDAC6 thus promoting ciliary reabsorption through deacetylation of axonemal tubulin [27]. 

Another family of proteins that appears to participate in the coordination of the ciliary and cell cycles is the NIMA-related protein kinases (Nrks or Neks). In the green algae *Chlamydomonas reinhardtii*, at least two different Neks have been shown to localize to flagella, regulate flagellar length and disassembly and participate in the G2/M transition and cell growth [28-30]. In mammals, there are 11 Neks and those that have been characterized have been shown to play a role in cell cycle regulation, and in some cases localize to the centrosome [31-33]. Further support for the link between the Neks and ciliary biology comes from studies demonstrating that mutations in *Nek1* and *Nek8* are the causal defect in two mouse models of polycystic kidney disease (PKD), a hallmark of the ciliopathies (Table **1**) and a phenotype characterized by a an altered balance between cell proliferation and differentiation [34, 35]. Nek1 localizes to centrosomes throughout the cell cycle and has been implicated in maintenance of the centrosome, formation of primary cilia and DNA damage checkpoint control and repair [36, 37]. In addition, Nek1 cycles through the nucleus and localize at discrete nuclear points after ionizing radiation-mediated DNA damage [38]. Thus, it could be possible that Nek1 interacts with members of the cell cycle machinery independently of its ciliary role. Nek8 localizes to the primary cilium during interphase and was undetectable during mitosis [39]. Interestingly, mutations in *NEK8* that affect its ciliary and centrosomal localization cause the cystic kidney disease Nephronophtisis type 9 [40]. Similarly, loss of Nek8 ciliary localization has been reported in primary kidney epithelial cells derived from mice models of PKD [41]. Despite its ciliary localization, Nek8 does not appear to be involved in cilium formation [39] but probably takes part in cilium signaling therefore likely affecting downstream events such as cell cycle progression. 

An indirect line of evidence supporting a cilia-related role for the Neks comes from phylogenetic analyses showing that this family of proteins is expanded in organisms that present cilia. Interestingly, the Neks seem to have co-evolved with those centrioles that function both in the context of centrosomes and basal bodies, leading the authors to suggest that Neks might play an important role in coordinating the function of centrioles, centrosomes and cilia [42, 43]. 

Several other proteins with well defined roles during cell division have been reported to localize to the primary cilium and/or participate in ciliogenesis (for some references see [44, 45]). However, the molecular mechanisms that underlie their relationship with the cilium are not well understood. 

## 3. CILIA-DEPENDENT CONTROL OF CELL PROLIFERATION AND DIFFERENTIATION

A question that remains open when thinking about the relationship between cilia and the cell cycle is what comes first, or in other words, who controls who? Is it signaling from the cell cycle machinery that triggers the assembly or disassembly of primary cilia, or signals from the cilium directly regulate cell cycle entry and/or progression? Although for the purpose of this review we have attempted to artificially separate these two scenarios, it is clear that at the cellular level the answer is both, and the final coordination of ciliogenesis and cell division is the result of a complex network of pathways and the activity of proteins both inside and outside of the cilium. In the previous section we have described that cell cycle associated proteins can influence the ability of a cell to ciliate. In the following sections we will describe how cilia and ciliary proteins can directly influence cell cycle progression and differentiation. 

### A. Cell Proliferation Regulated by Ciliary Proteins

#### Intraflagellar Transport Proteins

IFT is a motility process first described in *Chlamydomonas* by which large protein complexes are transported in and out of the cilium propelled by the activity of microtubule associated molecular motors [3]. This process is required during ciliogenesis and for the maintenance, function and re-absorption of primary cilia (for in depth reviews see Refs. [1, 4]). One IFT component that has been well characterized is Ift88/Polaris, a protein with documented roles in ciliary assembly, both *in vitro* and *in vivo *[46]. Most of the attention on this protein came from the realization that the *Chlamydomonas* *Ift88* gene is the ortholog of the mouse *Tg737*, the gene mutated in the Oak Ridge polycystic kidney (*orpk*) mouse [47, 48]. IFT88 is involved in IFT and localizes to the basal body and axoneme of primary cilia in quiescent mammalian ciliated cells [46, 49]. In proliferating cells, IFT88 is found in the centrosome in all stages of the cell cycle, both in ciliated and non-ciliated cell lines [50]. Interestingly, in HeLa cells under non-ciliated conditions, Robert and colleagues showed that IFT88/Polaris participate in restricting the G1/S transition, suggesting an extraciliary role of IFT88, independent of its participation in the IFT process [50]. This property of IFT88 appears to rely on its ability to interact with Che-1, a factor that promotes entrance into S phase through relieving the effect of the growth inhibitor factor retinoblastoma (Rb; Fig. **2**) [50, 51]. Overexpression of IFT88 destabilizes Che-1-Rb interaction promoting G1 arrest and conversely, knockdown of the protein favors cell proliferation. Since Che-1 and Rb are nuclear proteins, it is possible that IFT88 could be present in that subcellular compartment as well, increasing the complexity associated with ciliary proteins. Importantly, the capacity of centrosomal/ciliary proteins to enter the nucleus has been described for other proteins such as for example OFD1, a centrosomal protein mutated in the ciliopathy orofaciodigital syndrome type I (OMIM 311200) [52].

Several other examples support a direct role for IFT proteins in cell cycle regulation although the mechanisms have not been elucidated yet. For example, depleting IFT27 in *Chlamydomonas*, a small G-protein of the Rab family shown to be involved in IFT, results in cell growth inhibition, increased length of the cell cycle and defects in cytokinesis [53]. In mice, deletion of IFT20 specifically in kidney collecting duct cells produced abnormal orientation of the mitotic spindle, enhanced cell proliferation and cyst development [54]. However at this point it is not clear whether this effect on cell cycle is independent of the role of these IFT proteins in ciliary function. In both cases, decreased levels of the IFT protein resulted in ciliary defects and thus disturbed cilia-mediated signaling could be responsible, at least in part, for the observed effects on the cell cycle.

#### Polycystins: Cilia Dependent and Independent Control of Cell Proliferation 

The Polycystins (PC) are well studied ciliary proteins first discovered by their association with autosomal-dominant polycystic kidney disease (ADPKD), the most common form of hereditary kidney disease [55]. Mutations in *PKD1* and *PKD2*, which encode for PC1 and PC2 respectively, account for almost all ADPKD cases [56, 57]. PC1 is an integral membrane protein [58], an orphan G protein-coupled receptor involved in cell-cell and cell-matrix interactions while PC2 is a Ca^+2 ^permeable cation channel [59-61]. Both proteins interact through their C-terminal cytoplasmic tails and co-localize in the primary cilium where they are thought to work on a common pathway as mutations in either gene, whether in mice or humans, produce highly similar phenotypes [62-65]. Importantly, neither PC1 nor PC2 appear to be involved in cilia assembly or stability, as suggested by the observation that in human ADPKD kidneys or mouse kidneys with a *Pkd1* mutation, the primary cilia of cyst lining epithelial cells are neither absent nor shortened [55]. However, these proteins have a well documented role in regulating Ca^2+^ flux through ciliary-mediated mechanosensation (for some reviews see Refs. [55, 66, 67]).

PC1 and PC2 seem to be directly involved in the regulation of the cell cycle; the overall picture pointing to a role of these proteins in inhibiting cell proliferation in favor of cell differentiation. Overexpression of PC1 in kidney epithelial cells slows proliferation rates, protects from apoptosis and induces the spontaneous formation of branching tubules [68]. Some of these effects can be explained by the ability of PC1 to interact and activate JAK2 kinase and therefore the STAT pathway resulting in the upregulation of p21 and cell cycle arrest in G0/G1 (Fig. **2**) [69]. Moreover, this process requires PC2 and mutations that affect the interaction between PC1 and PC2 prevent the activation of the pathway. Also, PC2, together with PC1, was shown to regulate cell proliferation by directly interacting with the transcription factor Id2 and preventing its translocation into the nucleus (Fig.**2**) [70]. Id2 blocks the transcription of target genes such as p21, thus promoting cellular growth while inhibiting cell differentiation [71, 72]. Therefore, PC1/PC2 promote p21 expression and in that way inhibit cell proliferation. 

In the context of the cilium, PC1 and PC2 have been shown to mediate important sensory functions. Primary cilia act as mechanosensory organelles relaying mechanical cues into the cell that influence cellular processes such as proliferation and differentiation. It has been shown that movement of extracellular fluid can result in ciliary bending, for example in renal epithelial cells, which in turn triggers the opening of cilia located channels with the consequent increase in the intracellular Ca^2+^ concentration, a signal that is amplified by the additional release of Ca^2+^ from intracellular stores [73]. Importantly, PC1 and PC2 have been shown to be required in this process. The extracellular domain of PC1 is thought to act as a sensor of mechanical cues driving conformational changes in the protein that result in the activation of the PC2 channel, allowing Ca^2+^ entrance into the cell, thus regulating a number of calcium dependent downstream signaling events that control for example cell cycle entry and progression [64, 74, 75]. Therefore, ciliary proteins such as PC1 and PC2 can influence the balance between cell proliferation and differentiation possibly by both cilia dependent and independent mechanisms.

#### β-Arrestins

G protein-coupled receptors such as the somatostain type 3 receptor and smoothened, the receptor for Shh, accumulate at the primary cilium (see next section) [76, 77]. In turn, most of these types of receptors are regulated by the non-visual arrestins, β-arrestin1 and 2 [78, 79]. β-arrestin2 localizes to the cytoplasm and centrosomes of proliferating cells throughout the cell cycle whereas it is found in the axoneme of primary cilia in ciliated cells. β-arrestin2 has been shown to drive cells into G0 under the appropriate culture conditions and in that way it has been suggested to promote ciliation [80]. However, β-arrestin2 interacts with the IFT anterograde molecular motor Kif3a and thus a more direct role during ciliation cannot be ruled out. 

In summary, several proteins that localize to primary cilia or that are involved in ciliogenesis have been shown to restrict cell proliferation, either arresting cells at G1-S and/or at G2-M. In some cases, a partner known to be involved in cell cycle control has been shown to interact with these proteins raising the possibility that these moieties might play cilia-independent roles in cell cycle regulation. In other cases, the effect on the cell cycle could be a consequence of altering signaling pathways that operate trough the cilium and that control cell proliferation and differentiation. 

### B. Balancing Cell Proliferation and Differentiation Through Cilia-Mediated Paracrine Signaling

A number of studies have shown that primary cilia represent cellular organelles specialized in signal transduction that although continuous with the plasma membrane, present a distinct and specific composition. This separation is achieved by the formation of a diffusion barrier at the base of the cilium which has been shown to rely on the activity of Septin 2 (SEPT2). Depletion of SEPT2 has been shown to result in aberrant localization of ciliary proteins, defective cilia-dependent signaling and ciliogenesis [81]. This physical separation is critical to achieve the significant enrichment in receptors, ion-channels, adaptor proteins and transcription factors, among other moieties, that characterize primary cilia. In addition to this enrichment in signaling moieties, the primary cilium offers a smaller surface and volume than the cell body likely facilitating the interaction between the different molecules that are needed to integrate and transduce a given signaling cascade. Data gathered in recent years have promoted and supported this concept by showing that primary cilia play a key role in the coordination of diverse signaling pathways that control cell survival, proliferation and differentiation, and are therefore pivotal during development and for adult tissue homeostasis. In the following sections we will briefly review the role of primary cilia in paracrine signaling. 

#### Hedgehog (Hh) Signaling through the Cilium

**Fig. (3). The primary cilium in signal transduction.**
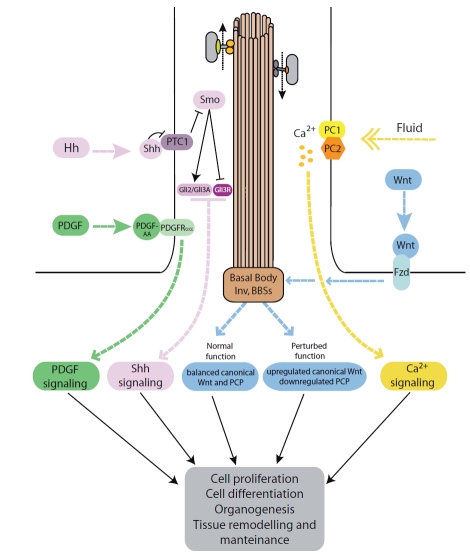
Schematic and simplified representation of the primary cilium and its role in the transduction of paracrine and Ca^2+^ signaling to ultimately control cellular homeostasis. Localization of PDGFRαα to the primary cilium is required for proper signaling upon activation by PDGF-AA (green). In Hh signaling, binding of Shh to PTC1 drives the translocation of the receptor outside of the primary cilium thus allowing the entry and accumulation of Smo in the ciliary compartment. This re-localization is required for the proper processing of the Gli transcription factors and is needed for correct Shh signaling (purple). The cilium and basal body proteins such as inversin/NPHP2 (Inv) and the BBSs are required for the modulation of Wnt signaling influencing the balance between canonical Wnt and PCP signaling (light blue). The ciliary localization of PC1 and PC2 is required to couple ciliary bending with Ca^2+^ signaling (yellow).

The Hh pathway regulates several important processes such as morphogenesis, patterning and growth, involving different tissue types and organs. In vertebrates there are three members of the Hh family of paracrine factors: Sonic (Shh), which is involved in central processes during embryonic and fetal development such as patterning of the neural tube and defining the anterior-posterior axis in the limb buds, Indian (Ihh) that is important in postnatal bone growth, and Desert (Dhh), which participates in spermatogenesis. Hh mediated signal transduction is complex and depends on several membrane-associated proteins, such as Patched-1 (Ptch) and Smoothened (Smo), and soluble transcription factors (Gli1, 2 and 3). In the absence of Hh signal, Gli1 is not expressed, Gli2 is degraded through the proteasome and Gli3 is proteolitically processed to a repressor form (Gli3R). Binding of the Hh factor to its receptor Ptch stimulates its endocytosis allowing Smo to reach the plasma membrane and cilium thus promoting the activation of the pathway by inhibiting both Gli-2 degradation and the production of Gli3R and favoring the formation of the activator form of Gli3 (Gli3A) (Fig. **3**). The net result is therefore the expression of Hh-target genes (reviewed in Ref. [82]). 

The first evidence that cilia were involved in Shh signaling in vertebrates was the observation that mutations in several genes coding for IFT related proteins (Ift172, Ift88 and Kif3a) produced phenotypes similar to those observed in *bona fide* s*hh* or *smo* mutant embryos, including open neural tube and limb and neural tube patterning defects [14]. More recent studies have shown that the dynamic localization of several components of the pathway to the ciliary compartment is required for proper signaling. It has been shown that Ptch, when localized to the cilium, is able to inhibit the entry of Smo into the organelle (Fig. **3**). Shh binding to Ptch1 results in the translocation of the receptor out of the cilium thus leading to an accumulation of Smo in the organelle in a process that requires the IFT motor Kif3a and the previously mentioned β-arrestins [83, 84]. Similarly, the Gli transcription factors Gli1, Gli2 and Gli3 have been shown to localize to primary cilia and their correct processing and function depends on intact IFT [12, 85]. 

Importantly, the correct separation between the ciliary compartment and the plasma membrane as well as the ciliary localization of Smo and other components of the pathway is required for proper Hh signaling [76, 81, 86]. Trafficking into the ciliary compartment appears to be required for Shh activation as evidenced in mice with congenital loss of Kif3a or Ift88, proteins required for anterograde IFT, which present phenotypes due to inactive Shh signaling [12, 13, 85]. Exogenously expressed Gli2 is unable to activate signaling in cells lacking anterograde IFT and Gli1 activity is indirectly affected because its expression is dependent on Hh signaling [12]. In contrast, impaired retrograde IFT appears to result in exacerbated Shh signaling [87, 88]. In the alien (aln) mouse, alterations in the ciliary protein THM1, the mouse ortholog of *C. reinhardtii* complex A protein Ift139, result in bulb-like structures in the tip of primary cilia due to impaired retrograde IFT. Smo and Gli proteins are sequestered in the cilium therefore resulting in the aberrant hyperactivation of the pathway [88]. Likewise, the retrograde IFT protein Ift122 has been shown to negatively regulate Shh signaling by differentially controlling the ciliary localization of both positive and negative regulators of the pathway. While the effectors Gli2 and Gli3 accumulate at the ciliary tips in Ift122 mutants, the inhibitor TULP3 is not found in the ciliary compartment [89]. However, generalizing that anterograde and retrograde transport will have opposite roles during Shh signaling is a simplification. For example, disruption of the dynein heavy chain Dync2h1, a protein with a role in retrograde IFT, results in the downregulation of the pathway [90]. Further support for the involvement of IFT in Hh signaling comes from a recent report showing that a hypomorphic allele of *Ift80*, an IFT component required for cilia formation and maintenance, resulted in a significant reduction in Hh pathway activation without loss or malformation of cilia [91], showing that it is not just the presence or absence of the organelle but rather an intact IFT machinery what is needed to accomplish the dynamic changes in cellular localization that modulate pathway activity. 

Although the majority of studies have been focused on Shh-mediated signaling, it is now known that primary cilia also participate in Ihh signaling. Ihh is required for the normal development of the long bones of the limbs through its activity regulating proliferation and differentiation of the chondrocyte lineage [92]. Conditional deletion of *Ift88* in limb mesenchyme produced shortening of the proximo-distal axis of the limbs, similarly to what is observed in *Ihh* mutants [92-94]. 

#### Signaling through Platelet-Derived Growth Factor Receptor Alpha (PDGFRαa)

The PDGF signaling pathway controls cell survival, proliferation and migration during development and in adult tissues and has also been shown to operate through the primary cilium (for an in depth review see Ref. [10]). Briefly, the PDGF family is composed by five homo- and heterodimers, synthesized and secreted by several different cell types. All isoforms operate through receptor tyrosine kinases, PDGFRα and PDGFRβ, which dimerize in three different homo- and heterodimer complexes: αα, αβ and ββ. The three dimeric PDGF receptor combinations transduce overlapping, but not identical, cellular signals. In particular, both αα- and ββ-receptor, distributed among different cell types, transduce potent mitogenic signals [95]. 

Initially, it was observed that PDGFRα is upregulated during serum starvation in cultured fibroblasts, a condition that also promotes ciliogenesis [95]. Indeed, PDGFRα localizes to the primary cilium in fibroblasts, in contrast to PGDRFβ that is present on the plasma membrane in both interphase and growth-arrested cells [96]. Importantly, it was shown that fibroblasts are able to respond to PDGF-AA, the specific ligand of PDGFRαα, only when cells are not proliferating, while activation of PDGFRββ occurred under both, proliferating and growth-arrested conditions. Ligand binding activates the PDGFR by promoting its dimerization and autophosphorylation on intracellular tyrosine residues thus serving as a docking site for SH2/PTB domain-containing adaptor and effector proteins. Two of these adaptor proteins are the Src homology Phosphotyrosyl Phosphatase (SHP2) and Phsophatidyl Inositol 3-OH Kinase (PI3K). The former promotes cell proliferation through the Ras-Raf-Mek1/2-Erk1/2 pathway while docking of PI3K to the activated receptor leads to the activation/generation of several downstream factors, including the multifunctional Akt protein kinase, able to activate mTORC1, a signaling complex involved in cellular growth and proliferation [97]. Stimulation of PDGFRαα in quiescent fibroblasts produced activation of Mek1/2, Erk1/2 and Akt, together with Rb and Cdc2 phosphorylation, which indicates cell cycle entry [96]. Consistent results were obtained with mouse embryonic fibroblasts (MEF) obtained from wild type and *Ift88*/*Tg737^orpk ^*mutant mice. In MEFs from *Tg737^orpk^* mice the levels of PDGFRα were not upregulated in response to serum starvation and PDGF-AA was unable to induce cells to re-enter the cell cycle. In contrast, PDGF-BB-mediated activation of PDGFRβ was unaffected and cells re-entered the cell cycle after PDGF-BB or serum stimulation [96].

In summary, the ciliary localization of PDGFRαα implies that the cell will be able to respond to signals that operate through this receptor, only in the presence of the organelle, i.e. under growth arrested-conditions. Moreover, as receptor stimulation pushes the cell to resume the cell cycle, the cell response will also trigger disassembly of the cilium, and in that way, it will become unresponsive to PDGFRαα signaling. However, the molecular actors that connect PDGFRαα stimulation and cilia disassembly have not been established. Christensen and colleagues have proposed that PDGFRαa activation could lead to the sequential activation of Crk, HEF-1 and AurA, the latter leading to ciliary disassembly [10]. 

#### Ciliary Modulation of Wnt Outcome: Keeping the Balance Between Canonical Wnt and PCP

The Wnt signaling cascade is another central pathway involved in the regulation of cell proliferation, differentiation and thus central during development. Depending on a number of factors, the pathway can activate a series of distinct effectors that result in different biological outcomes (for a review on the pathway see Ref. [98]). The canonical Wnt signaling cascade relies on the β-catenin mediated transcriptional activation of a number of TCF-LEF1 responsive genes that function during development controlling proliferation, cell cycle progression and differentiation [99]. Another important output of Wnt signaling is the PCP pathway (Planar Cell Polarity) responsible for providing positional clues that are required to coordinate multicellular processes such as concerted movements during early developmental stages and to correctly organize and orient cells in the plane of a given tissue [100, 101]. In recent years, several studies have indicated an active role of the cilium and ciliary proteins in the transduction and modulation of this signaling cascade thus providing another way through which this organelle can influence cellular homeostasis.

Briefly, the Wnts are secreted factors that activate the pathway by binding Frizzled receptors. It is a combination of the specific Wnt molecule, the receptor and the activity of proteins such as Disheveled (Dvl) what determines the specific Wnt cascade that is activated in each case. Dvl represses the β-catenin destruction complex composed of GSK3bβ, APC and axin. Nuclear localization of Dvl results in accumulation of β-catenin and thus the activation of the canonical Wnt signaling cascade. In contrast, when Dvl is localized to the plasma membrane, the PCP pathway is activated (for reviews see Refs. [11, 101]). 

Important insight into the link between cilia/basal bodies and Wnt regulation came from studies by Simons and colleagues demonstrating that inversin (Inv), the protein mutated in Nephronophthisis type 2 (NPHP2), interacts with Dvl targeting it for degradation [102]. The authors showed that mutations in NPHP2/inversin result in upregulation of canonical Wnt and importantly the concomitant downregulation of PCP [102]. Further support for the link between cilia and Wnt came from studies in the ciliopathy Bardet-Biedl syndrome (BBS; OMIM 209900). The BBS proteins characterized so far (16 identified to date) localize to centrosomes and basal bodies of cilia [103-107]. In addition, several BBS proteins have been found in a complex, termed BBSome that plays a role during ciliogenesis, recognizing sorting signals in different ciliary proteins and thus actively participating in translocating these moieties into the ciliary compartment [108-110]. Similarly, BBS3, which has not been found associated with the BBSome, localizes to the distal end of the basal body likely playing a role during ciliogenesis controlling traffic into and out of the cilium [111].

In cultured cells, depletion of different BBS proteins and the IFT molecular motor Kif3a resulted in the upregulation of canonical Wnt signaling [112]. Similarly, upregulation of canonical Wnt is also observed in the *orpk* mouse model as well as in *Kif3a^-/-^* and *Ofd1^-/-^*animals [113]. Interestingly, depletion of different BBS proteins in both zebrafish and mouse models result in typical PCP phenotypes such as convergence and extension defects during gastrulation, misoriented stereociliary bundles in the cochlea and open neural tubes. In addition, different BBS genes have been shown to genetically interact with core PCP genes [104, 114, 115]. More recently, it has been shown that mutations in the PCP gene *Fritz* are found both in BBS and the related ciliopathy Meckel Gruber syndrome (MKS; OMIM 249000), further linking the BBS proteins with PCP regulation [116]. Fritz controls the localization of septins, cytoskeletal proteins involved in cell proliferation and migration, and was shown to play a role both during concerted cell movements and ciliogenesis in *Xenopus *embryos [116]. Therefore, cilia appear to constrain canonical Wnt signaling and molecules such as inversin and the BBS proteins participate in balancing the two main outcomes of the Wnt pathway (Fig. **3**). More recently, Wiens and colleagues have shown that in contrast to other BBS proteins, overexpression of BBS3 results in upregulated canonical Wnt signaling. However, they also showed that increased levels of BBS3 result in a reduction in the number of ciliated cells in culture, thus further suggesting that cilia are indeed able to keep canonical Wnt in check [111].

Some recent reports however are posing the question of whether Wnt regulation is dependent on the primary cilium as an organelle or whether is the result of the activity of particular ciliary proteins such as the BBS, either in the cilium or potentially outside of it. Zebrafish mutants that lack cilia due to mutations in *ift88* have been shown to preserve normal Wnt signaling responses, both canonical and PCP, while hedgehog signaling is misregulated [117]. Similarly, mouse embryos and cells where primary cilia are affected by the loss of Kif3a, Ift88 or Ift172 have been shown to normally express the canonical Wnt target Axin2, activate a canonical Wnt reporter gene to levels that are comparable with controls and respond to the ligand Wnt3a similarly to controls [118]. Therefore, although several studies have linked the Wnt pathway with the primary cilium, there are conflictive data and a lack of consensus as to what extent cilia participate in Wnt signal transduction. While we have summarized different lines of evidence both in favor and against the cilia-Wnt link, we recommend a review by Wallingford and Mitchell for a thoughtful in depth discussion on the topic [119]. 

## 4. PHENOTYPIC OUTCOME OF THE CILIA-CELL CYCLE LINK: THE CILIOPATHIES AND CANCER

As exemplified along the review, primary cilia are key organelles at the interface between cells and their environment, sensing, integrating and relaying a host of mechanical and chemical cues to finally modulate cell fate. In particular we have discussed the participation of this organelle and its associated proteins in the regulation of cell proliferation and differentiation. Therefore, questions that arise are whether the link between cilia and the cell cycle is reflected in the ciliopathies, and on the other hand, whether a proliferative disorder, such as cancer, is characterized by ciliary dysfunction. 

To date, we recognize a series of specific phenotypes such as the formation of cysts in the kidney and liver, retinal degeneration, obesity, polydactyly, *situs* abnormalities, central nervous system malformations as typical outcomes of ciliary dysfunction and thus shared, to a variable extent, among the different ciliopathies (Table **1**). Several of these phenotypes can be clearly seen as a misregulation of the balance between cell proliferation and differentiation. One of the best examples is the development of cysts in the kidney, the pathological hallmark of PKD and a highly prevalent phenotype among all the ciliopathies. In fact, different therapeutic interventions currently being tested target primarily the increased cell proliferation aspect of the disorder. For example, Bukanov and colleagues have shown that treatment of two animal models of PKD, the jck and the cpk mouse, with the cyclin-dependent kinase inhibitor roscovitine results in the amelioration of the cystic phenotype [120]. In a simplified view, primary cilia could be integrating mechanical cues, such as fluid flow in the renal tubules, and the activity of different signaling cascades, to therefore coordinate cell cycle entry/exit, favoring differentiation in detriment of cell proliferation [67].

Although it is clear that ciliary dysfunction can result in dysplastic processes, typical proliferative disorders such as cancer have not been extensively associated with the ciliopathies. However, recent reports are indeed linking primary cilia with cancer. For example, Seeley and colleagues have shown that ciliogenesis is lost specifically in pancreatic ductal adenocarcinoma cancer cells, even under non-proliferating conditions. Importantly, inhibiting Kras signaling resulted in the restoration of ciliation leading the authors to suggest that Kras might be directly inhibiting the formation of primary cilia [121]. Similarly, sporadic clear renal cell carcinoma, characterized by mutations in the tumor suppressor VHL, a protein thought to play a role in cilia maintenance, also presents a significantly reduced percentage of ciliated cells [122]. 

These cases might therefore illustrate the perhaps intuitive view that cilia restrain and are incompatible with cell proliferation. However, cilia have been shown to be required to maintain and expand certain cell populations and for the progression of certain cancer types such as those dependent on Shh signaling. Shh has been shown to be needed to maintain and expand granule cell precursors in the cerebellum and depletion of *Ift88*, *Kif3a* or *Smo* in these cells results in cerebellar hypoplasia [123, 124]. Interestingly, aberrant Shh signaling has been shown to play a pivotal role in the development of medulloblastoma. Importantly, Han and colleagues have shown that ablation of primary cilia block the formation of this type of tumor when it is driven by constitutively active Smo further supporting that Shh signaling relies on an intact cilium. However, tumor progression was promoted in the absence of cilia when it was driven by active Gli2 [125]. Similarly, basal cell carcinoma progression is promoted or inhibited by ciliary function depending on whether is caused by activated Smo or Gli2 respectively [9]. These data shows that the presence or absence of an intact cilium can have radically different consequences depending on a number of other factors such as whether the initiating oncogenic alteration is upstream or downstream of the organelle [125, 126]. Therefore, a “tumor suppressor” view of the primary cilium is likely a simplification of the complex biological role of the organelle, and explains, at least in part, the overall lack of cancer phenotypes as *bona fide* ciliopathy associated clinical manifestations. 

## 5. CONCLUDING REMARKS

The last decade has seen an impressive amount of work on primary cilia, likely fueled by the realization that this organelle plays a key role in the pathogenesis of a number of human conditions. We have shown examples of multiple ways by which cilia can participate or modulate cell fate decisions thus providing important insight to understand the cellular basis of the different phenotypes and conditions that characterize the ciliopathies (for an in depth review see for example Ref. [2]). However, our knowledge on the biology of cilia is likely far from complete. For example, a combination of bioinformatic, expression profiling, proteomic and genomic studies performed by multiple groups has led to the construction of a ciliary proteome (Ref. [127] and references within). While this dataset contains a list of proteins enriched for moieties known to be involved in the formation, maintenance and function of cilia, it also includes a significant number of proteins of unknown biological function. Thus, cilia might be playing additional biological roles that are not well understood to this date. Furthermore, in addition to their role on Wnt, Hh and PDGF signaling, cilia are being shown to be involved in other signaling cascades. It has recently been shown that cilia actively participate in the regulation of mTOR (mammalian Target Of Rapamicyn), a signaling network with a central role in the maintenance of cellular homeostasis by integrating stimuli such as the availability of nutrients with cell responses that determine growth and proliferation [97]. Boehlke and colleagues have recently shown that bending of the primary cilium is required to downregulate the activity of mTOR and therefore affects the control of cell size, one of the outcomes of the pathway. Interestingly, their data show that the role of the cilium in this process appears to rely on the activity of the tumor suppressor LKB1, which localizes to primary cilia and appears to mediate AMPK phosphorylation at the basal body in response to fluid flow thus providing another example of how cilia can help integrate mechanical stimuli with cellular responses [128]. Therefore, as we try to assess the impact of ciliary dysfunction at the cellular and organism level, it will be critical to fully realize and dissect the complexity of this organelle, obtaining a complete list of the multiple biological processes that directly or indirectly depend on its integrity. In addition, we will need to start dissecting the role of the cilium both at different time-points during development and on different cell types in order to address the question of why are different tissues and organs affected differently by ciliary dysfunction. Lastly we will need to understand the role of ciliary proteins both in the context of the organelle as well as outside of it, a problem that perhaps has been overshadowed by the impressive recent advances in the field of ciliary biology. 

## Figures and Tables

**Table 1.** Different Cilia Associated Phenotypes and Examples of Ciliopathies

	PKD	NPHP	SLSN	EVC	JAth	OFD	ALMS	JS	BBS	MKS
Cystic kidney	√	√	√		√	√	√	√	√	√
CNS malformations				√	√	√	√	√	√	√
Retinal degeneration			√		√		√	√	√	
*Situs* defects		√	√		√			√	√	√
Polydactyly				√	√	√		√	√	√
Gonadal malformations				√					√	√
Heart disease				√			√		√	
Mental retardation				√	√	√		√	√	√
Obesity							√		√	
Diabetes							√		√	
Skeletal defects				√	√	√				

PKD: Polycystic kidney disease; NPHP: nephronophthisis (OMIM 256100); SLSN: Senior-Løken Syndrome (OMIM 266900); EVC: Ellis van Creveld (OMIM 225500); JATD: Jeune asphyxiating thoracic dystrophy (OMIM 208500); OFD: Orofaciodigital syndrome; ALMS: Alström syndrome (OMIM 203800); JS: Joubert Syndrome/Cerebello-oculo-renal syndrome (OMIM 213300); BBS: Bardet-Biedl syndrome; MKS: Meckel-Gruber syndrome; CNS: Central nervous system.

## References

[1] Rosenbaum JL, Witman GB (2002). Intraflagellar transport. Nat. Rev. Mol. Cell Biol.

[2] Cardenas-Rodriguez M, Badano JL (2009). Ciliary biology: understanding the cellular and genetic basis of human ciliopathies. Am. J. Med. Genet. C Semin. Med. Genet.

[3] Kozminski KG, Johnson KA, Forscher P, Rosenbaum JL (1993). A motility in the eukaryotic flagellum unrelated to flagellar beating. Proc. Natl. Acad. Sci. USA.

[4] Pedersen LB, Rosenbaum JL (2008). Intraflagellar transport (IFT) role in ciliary assembly, resorption and signalling. Curr. Top. Dev. Biol.

[5] Badano JL, Mitsuma N, Beales PL, Katsanis N (2006). The ciliopathies: An emergin class of human genetic disorders. Annu. Rev. Genomics Hum. Genet.

[6] Fliegauf M, Benzing T, Omran H (2007). When cilia go bad: cilia defects and ciliopathies. Nat. Rev. Mol. Cell. Biol.

[7] Gerdes JM, Davis EE, Katsanis N (2009). The vertebrate primary cilium in development, homeostasis, and disease. Cell.

[8] Sharma N, Berbari NF, Yoder BK (2008). Ciliary dysfunction in developmental abnormalities and diseases. Curr. Top. Dev. Biol.

[9] Wong SY, Seol AD, So PL, Ermilov AN, Bichakjian CK, Epstein EHJ, Dlugosz AA, Reiter JF (2009). Primary cilia can both mediate and suppress Hedgehog pathway-dependent tumorigenesis. Nat. Med.

[10] Christensen ST, Pedersen SF, Satir P, Veland IR, Schneider L (2008). The primary cilium coordinates signaling pathways in cell cycle control and migration during development and tissue repair. Curr. Top. Dev. Biol.

[11] Gerdes JM, Katsanis N (2008). Ciliary function and Wnt signal modulation. Curr. Top. Dev. Biol.

[12] Haycraft CJ, Banizs B, Aydin-Son Y, Zhang Q, Michaud EJ, Yoder BK (2005). Gli2 and Gli3 localize to cilia and require the intraflagellar transport protein polaris for processing and function. PLoS Genet.

[13] Huangfu D, Anderson KV (2005). Cilia and Hedgehog responsiveness in the mouse. Proc. Natl. Acad. Sci. USA.

[14] Huangfu D, Liu A, Rakeman AS, Murcia NS, Niswander L, Anderson KV (2003). Hedgehog signalling in the mouse requires intraflagellar transport proteins. Nature.

[15] Debec A, Sullivan W, Bettencourt-Dias M (2010). Centrioles active players or passengers during mitosis?. Cell. Mol. Life Sci.

[16] Hinchcliffe EH, Li C, Thompson EA, Maller JL, Sluder G (1999). Requirement of Cdk2-cyclin E activity for repeated centrosome reproduction in Xenopus egg extracts. Science.

[17] Plotnikova OV, Pugacheva EN, Golemis EA (2009). Primary cilia and the cell cycle. Methods Cell Biol.

[18] Santos N, Reiter JF (2008). Building it up and taking it down: the regulation of vertebrate ciliogenesis. Dev. Dyn.

[19] Rieder CL, Jensen CG, Jensen LC (1979). The resorption of primary cilia during mitosis in a vertebrate (PtK1) cell line. J. Ultrastruct. Res.

[20] Basto R, Lau J, Vinogradova T, Gardiol A, Woods CG, Khodjakov A, Raff JW (2006). Flies without centrioles. Cell.

[21] Bettencourt-Dias M, Rodrigues-Martins A, Carpenter L, Riparbelli M, Lehmann L, Gatt MK, Carmo N, Balloux F, Callaini G, Glover DM (2005). SAK/PLK4 is required for centriole duplication and flagella development. Curr. Biol.

[22] Habedanck R, Stierhof YD, Wilkinson CJ, Nigg EA (2005). The Polo kinase Plk4 functions in centriole duplication. Nat. Cell Biol.

[23] Uetake Y, Loncarek J, Nordberg JJ, English CN, La Terra S, Khodjakov A, Sluder G (2007). Cell cycle progression and de novo centriole assembly after centrosomal removal in untransformed human cells. J. Cell Biol.

[24] Chen Z, Indjeian VB, McManus M, Wang L, Dynlacht BD (2002). CP110 a cell cycle-dependent CDK substrate, regulates centrosome duplication in human cells. Dev. Cell.

[25] Spektor A, Tsang WY, Khoo D, Dynlacht BD (2007). Cep97 and CP110 suppress a cilia assembly program. Cell.

[26] Tsang WY, Bossard C, Khanna H, Peränen J, Swaroop A, Malhotra V, Dynlacht BD (2008). CP110 suppresses primary cilia formation through its interaction with CEP290, a protein deficient in human ciliary disease. Dev. Cell.

[27] Pugacheva EN, Jablonski SA, Hartman TR, Henske EP, Golemis EA (2007). HEF1-dependent Aurora A activation induces disassembly of the primary cilium. Cell.

[28] Bradley BA, Quarmby LM (2005). A NIMA-related kinase, Cnk2p, regulates both flagellar length and cell size in Chlamydomonas. J. Cell Sci.

[29] Mahjoub MR, Montpetit B, Zhao L, Finst RJ, Goh B, Kim AC, Quarmby LM (2002). The FA2 gene of Chlamydomonas encodes a NIMA family kinase with roles in cell cycle progression and microtubule severing during deflagellation. J. Cell Sci.

[30] Wloga D, Camba A, Rogowski K, Manning G, Jerka-Dziadosz M, Gaertig J (2006). Members of the NIMA-related kinase family promote disassembly of cilia by multiple mechanisms. Mol. Biol. Cell.

[31] Tan BC, Lee SC (2004). Nek9 a novel FACT-associated protein, modulates interphase progression. J. Biol. Chem.

[32] Yin MJ, Shao L, Voehringer D, Smeal T, Jallal B (2003). The serine/threonine kinase Nek6 is required for cell cycle progression through mitosis. J. Biol. Chem.

[33] Yissachar N, Salem H, Tennenbaum T, Motro B (2006). Nek7 kinase is enriched at the centrosome, and is required for proper spindle assembly and mitotic progression. FEBS Lett.

[34] Liu S, Lu W, Obara T, Kuida S, Lehoczky J, Dewar K, Drummond IA, Beier DR (2002). A defect in a novel Nek-family kinase causes cystic kidney disease in the mouse and in zebrafish. Development.

[35] Upadhya P, Birkenmeier EH, Birkenmeier CS, Barker JE (2000). Mutations in a NIMA-related kinase gene, Nek1, cause pleiotropic effects including a progressive polycystic kidney disease in mice. Proc. Natl. Acad. Sci. USA.

[36] Shalom O, Shalva N, Altschuler Y, Motro B (2008). The mammalian Nek1 kinase is involved in primary cilium formation. FEBS Lett.

[37] White MC, Quarmby LM (2008). The NIMA-family kinase, Nek1 affects the stability of centrosomes and ciliogenesis. BMC Cell Biol.

[38] Hilton LK, White MC, Quarmby LM (2009). The NIMA-related kinase NEK1 cycles through the nucleus. Biochem. Biophys. Res. Commun.

[39] Mahjoub MR, Trapp ML, Quarmby LM (2005). NIMA-related kinases defective in murine models of polycystic kidney diseases localize to primary cilia and centrosomes. J. Am. Soc. Nephrol.

[40] Otto EA, Trapp ML, Schultheiss UT, Helou J, Quarmby LM, Hildebrandt F (2008). NEK8 mutations affect ciliary and centrosomal localization and may cause nephronophthisis. J. Am. Soc. Nephrol.

[41] Smith LA, Bukanov NO, Husson H, Russo RJ, Barry TC, Taylor AL, Beier DR, Ibraghimov-Beskrovnaya O (2006). Development of polycystic kidney disease in juvenile cystic kidney mice: insights into pathogenesis, ciliary abnormalities, and common features with human disease. J. Am. Soc. Nephrol.

[42] Parker JD, Bradley BA, Mooers AO, Quarmby LM (2007). Phylogenetic analysis of the Neks reveals early diversification of ciliary-cell cycle kinases. PloS ONE.

[43] Quarmby LM, Mahjoub MR (2005). Caught Nek-ing: cilia and centrioles. J. Cell Sci.

[44] Jurczyk A, Gromley A, Redick S, San Agustin J, Witman G, Pazour GJ, Peters DJ, Doxsey S (2004). Pericentrin forms a complex with intraflagellar transport proteins and polycystin-2 and is required for primary cilia assembly. J. Cell Biol.

[45] Patzke S, Redick S, Warsame A, Murga-Zamalloa CA, Khanna H, Doxsey S, Stokke T (2010). CSPP is a ciliary protein interacting with Nephrocystin 8 and required for cilia formation. Mol. Biol. Cell.

[46] Pazour GJ, Dickert BL, Vucica Y, Seeley ES, Rosenbaum JL, Witman GB, Cole DG (2000). Chlamydomonas IFT88 and its mouse homologue, polycystic kidney disease gene tg737, are required for assembly of cilia and flagella. J. Cell Biol.

[47] Moyer JH, Lee-Tischler MJ, Kwon HY, Schrick JJ, Avner ED, Sweeney WE, Godfrey VL, Cacheiro NL, Wilkinson JE, Woychik RP (1994). Candidate gene associated with a mutation causing recessive polycystic kidney disease in mice. Science.

[48] Yoder BK, Richards WG, Sweeney WE, Wilkinson JE, Avener ED, Woychik RP (1995). Insertional mutagenesis and molecular analysis of a new gene associated with polycystic kidney disease. Proc. Assoc. Am. Physicians.

[49] Taulman PD, Haycraft CJ, Balkovetz DF, Yoder BK (2001). Polaris, a protein involved in left-right axis patterning, localizes to basal bodies and cilia. Mol. Biol. Cell.

[50] Robert A, Margall-Ducos G, Guidotti JE, Brégerie O, Celati C, Bréchot C, Desdouets C (2007). The intraflagellar transport component IFT88/polaris is a centrosomal protein regulating G1-S transition in non-ciliated cells. J. Cell Sci.

[51] Fanciulli M, Bruno T, Di Padova M, De Angelis R, Iezzi S, Iacobini C, Floridi A, Passananti C (2000). Identification of a novel partner of RNA polymerase II subunit 11, Che-1, which interacts with and affects the growth suppression function of Rb. FASEB J.

[52] Giorgio G, Alfieri M, Prattichizzo C, Zullo A, Cairo S, Franco B (2007). Functional characterization of the OFD1 protein reveals a nuclear localization and physical interaction with subunits of a chromatin remodeling complex. Mol. Biol. Cell.

[53] Qin H, Wang Z, Diener D, Rosenbaum J (2007). Intraflagellar transport protein 27 is a small G protein involved in cell-cycle control. Curr. Biol.

[54] Jonassen JA, San Agustin J, Follit JA, Pazour GJ (2008). Deletion of IFT20 in the mouse kidney causes misorientation of the mitotic spindle and cystic kidney disease. J. Cell Biol.

[55] Zhou J (2009). Polycystins and primary cilia: primers for cell cycle progression. Annu. Rev. Physiol.

[56] Consortium TEPKD (1994). The polycystic kidney disease 1 gene encodes a 14 kb transcript and lies within a duplicated region on chromosome 16. Cell.

[57] Mochizuki T, Wu G, Hayashi T, Xenophontos S, Veldhuisen B, Saris J, Renolds D, Cai Y, Gabow P, Pierides A, Kimberling W, Breuning M, Deltas C, Peters D, Somlo S (1996). PKD2, a gene for polycystic kidney disease that encodes an integral membrane protein. Science.

[58] Consortium TIPKD (1995). Polycystic kidney disease: the complete structure of the PKD1 gene and its protein. The International Polycystic Kidney Disease Consortium. Cell.

[59] Delmas P, Nomura H, Li X, Lakkis M, Luo Y, Segal Y, Fernandez-Fernandez JM, Harris P, Frischauf AM, Brown DA, Zhou J (2002). Constitutive activation of G-proteins by polycystin-1 is antagonized by polycystin-2. J. Biol. Chem.

[60] Parnell SC, Magenheimer BS, Maser RL, Zien CA, Frischauf AM, Calvet JP (2002). Polycystin-1 activation of c-Jun N-terminal kinase and AP-1 is mediated by heterotrimeric G proteins. J. Biol. Chem.

[61] Gonzalez-Perrett S, Kim K, Ibarra C, Damiano AE, Zotta E, Batelli M, Harris PC, Reisin IL, Arnaout MA, Cantiello HF (2001). Polycystin-2, the protein mutated in autosomal dominant polycystic kidney disease (ADPKD), is a Ca^2+^-permeable nonselective cation channel. Proc. Natl. Acad. Sci. USA.

[62] Pazour GJ, San Agustin JT, Follit JA, Rosenbaum JL, Witman GB (2002). Polycystin-2 localizes to kidney cilia and the ciliary level is elevated in ORPK mice with polycystic kidney disease. Curr. Biol.

[63] Qian F, Germino FJ, Cai Y, Zhang X, Somlo S, Germino GG (1997). PKD1 interacts with PKD2 through a probable coiled-coil domain. Nat. Genet.

[64] Tsiokas L, Kim E, Arnould T, Sukhatme VP, Walz G (1997). Homo- and heterodimeric interactions between the gene products of *PKD1* and *PKD2*. Proc. Natl. Acad. Sci. USA.

[65] Yoder BK, Hou X, Guay-Woodford LM (2002). The polycystic kidney disease proteins, polycystin-1, polycystin-2, polaris, and cystin, are co-localized in renal cilia. J. Am. Soc. Nephrol.

[66] Delmas P (2004). Polycystins: from mechanosensation to gene regulation. Cell.

[67] Gascue C, Katsanis N, Badano JL (2010). Cystic diseases of the kidney: ciliary dysfunction and cystogenic mechanisms. Pediatr. Nephrol.

[68] Boletta A, Qian F, Onuchic LF, Bhunia AK, Phakdeekitcharoen B, Hanaoka K, Guggino W, Monaco L, Germino GG (2000). Polycystin-1, the gene product of PKD1, induces resistance to apoptosis and spontaneous tubulogenesis in MDCK cells. Mol. Cell.

[69] Bhunia AK, Piontek K, Boletta A, Liu L, Qian F, Xu PN, Germino FJ, Germino GG (2002). PKD1 induces p21(waf1) and regulation of the cell cycle *via* direct activation of the JAK-STAT signaling pathway in a process requiring PKD2. Cell.

[70] Li X, Luo Y, Starremans PG, McNamara CA, Pei Y, Zhou J (2005). Polycystin-1 and polycystin-2 regulate the cell cycle through the helix-loop-helix inhibitor Id2. Nat. Cell Biol.

[71] Peverali FA, Ramqvist T, Saffrich R, Pepperkok R, Barone MV, Philipson L (1994). Regulation of G1 progression by E2A and Id helix-loop-helix proteins. Embo J.

[72] Prabhu S, Ignatova A, Park ST, Sun XH (1997). Regulation of the expression of cyclin-dependent kinase inhibitor p21 by E2A and Id proteins. Mol. Cell Biol.

[73] Praetorius HA, Spring KR (2001). Bending the MDCK cell primary cilium increases intracellular calcium. J. Membr. Biol.

[74] Hanaoka K, Qian F, Boletta A, Bhunia AK, Piontek K, Tsiokas L, Sukhatme VP, Guggino WB, Germino GG (2000). Co-assembly of polycystin-1 and -2 produces unique cation-permeable currents. Nature.

[75] Nauli SM, Alenghat FJ, Luo Y, Williams E, Vassilev P, Li X, Elia AE, Lu W, Brown EM, Quinn SJ, Ingber DE, Zhou J (2003). Polycystins 1 and 2 mediate mechanosensation in the primary cilium of kidney cells. Nat. Genet.

[76] Corbit KC, Aanstad P, Singla V, Norman AR, Stainier DY, Reiter JF (2005). Vertebrate Smoothened functions at the primary cilium. Nature.

[77] Handel M, Schulz S, Stanarius A, Schreff M, Erdtmann-Vourliotis M, Schmidt H, Wolf G, Hollt V (1999). Selective targeting of somatostatin receptor 3 to neuronal cilia. Neuroscience.

[78] DeWire SM, Ahn S, Lefkowitz RJ, Shenoy SK (2007). Beta-arrestins and cell signaling. Annu. Rev. Physiol.

[79] Moore CA, Milano SK, Benovic JL (2007). Regulation of receptor trafficking by GRKs and arrestins. Annu. Rev. Physiol.

[80] Molla-Herman A, Boularan C, Ghossoub R, Scott MG, Burtey A, Zarka M, Saunier S, Concordet JP, Marullo S, Benmerah A (2008). Targeting of beta-arrestin2 to the centrosome and primary cilium: role in cell proliferation control. PLoS One.

[81] Hu Q, Milenkovic L, Jin H, Scott MP, Nachury MV, Spiliotis ET, Nelson WJ (2010). A septin diffusion barrier at the base of the primary cilium maintains ciliary membrane protein distribution. Science.

[82] Varjosalo M, Taipale J (2008). Hedgehog: functions and mechanisms. Genes Dev.

[83] Kovacs JJ, Whalen EJ, Liu R, Xiao K, Kim J, Chen M, Wang J, Chen W, Lefkowitz RJ (2008). Beta-arrestin-mediated localization of smoothened to the primary cilium. Science.

[84] Rohatgi R, Milenkovic L, Scott MP (2007). Patched1 regulates hedgehog signaling at the primary cilium. Science.

[85] Liu A, Wang B, Niswander LA (2005). Mouse intraflagellar transport proteins regulate both the activator and repressor functions of Gli transcription factors. Development.

[86] Aanstad P, Santos N, Corbit KC, Scherz PJ, Trinh LA, Salvenmoser W, Huisken J, Reiter JF, Stainier DY (2009). The Extracellular Domain of Smoothened Regulates Ciliary Localization and Is Required for High-Level Hh Signaling. Curr. Biol.

[87] Stottmann RW, Tran PV, Turbe-Doan A, Beier DR (2009). Ttc21b is required to restrict sonic hedgehog activity in the developing mouse forebrain. Dev. Biol.

[88] Tran PV, Haycraft CJ, Besschetnova TY, Turbe-Doan A, Stottmann RW, Herron BJ, Chesebro AL, Qiu H, Scherz PJ, Shah JV, Yoder BK, Beier DR (2008). THM1 negatively modulates mouse sonic hedgehog signal transduction and affects retrograde intraflagellar transport in cilia. Nat. Genet.

[89] Qin J, Lin Y, Norman RX, Ko HW, Eggenschwiler JT (2011). Intraflagellar transport protein 122 antagonizes Sonic Hedgehog signaling and controls ciliary localization of pathway components. Proc. Natl. Acad. Sci. USA.

[90] Ocbina PJ, Anderson KV (2008). Intraflagellar transport, cilia, and mammalian Hedgehog signaling: analysis in mouse embryonic fibroblasts. Dev. Dyn.

[91] Rix S, Calmont A, Scambler PJ, Beales PL (2011). An Ift80 mouse model of short rib polydactyly syndromes shows defects in hedgehog signalling without loss or malformation of cilia. Hum. Mol. Genet.

[92] St-Jacques B, Hammerschmidt M, McMahon AP (1999). Indian hedgehog signaling regulates proliferation and differentiation of chondrocytes and is essential for bone formation. Genes Dev.

[93] Haycraft CJ, Zhang Q, Song B, Jackson WS, Detloff PJ, Serra R, Yoder BK (2007). Intraflagellar transport is essential for endochondral bone formation. Development.

[94] Karp SJ, Schipani E, St-Jacques B, Hunzelman J, Kronenberg H, McMahon AP (2000). Indian hedgehog coordinates endochondral bone growth and morphogenesis *via* parathyroid hormone related-protein-dependent and -independent pathways. Development.

[95] Andrae J, Gallini R, Betsholtz C (2008). Role of platelet-derived growth factors in physiology and medicine. Genes Dev.

[96] Schneider L, Clement CA, Teilmann SC, Pazour GJ, Hoffmann EK, Satir P, Christensen ST (2005). PDGFRαlphaalpha Signaling Is Regulated through the Primary Cilium in Fibroblasts. Curr. Biol.

[97] Zoncu R, Efeyan A, Sabatini DM (2011). mTOR: from growth signal integration to cancer, diabetes and ageing. Nat. Rev. Mol. Cell. Biol.

[98] Logan CY, Nusse R (2004). The Wnt signaling pathway in development and disease. Annu. Rev. Cell Dev. Biol.

[99] Grigoryan T, Wend P, Klaus A, Birchmeier W (2008). Deciphering the function of canonical Wnt signals in development and disease: conditional loss- and gain-of-function mutations of beta-catenin in mice. Genes Dev.

[100] Fanto M, McNeill H (2004). Planar polarity from flies to vertebrates. J. Cell Sci.

[101] Veeman MT, Axelrod JD, Moon RT (2003). A second canon. Functions and mechanisms of beta-catenin-independent Wnt signaling. Dev. Cell.

[102] Simons M, Gloy J, Ganner A, Bullerkotte A, Bashkurov M, Kronig C, Schermer B, Benzing T, Cabello OA, Jenny A, Mlodzik M, Polok B, Driever W, Obara T, Walz G (2005). Inversin, the gene product mutated in nephronophthisis type II, functions as a molecular switch between Wnt signalling pathways. Nat. Genet.

[103] Ansley SJ, Badano JL, Blacque OE, Hill J, Hoskins BE, Leitch CC, Kim JC, Ross AJ, Eichers ER, Teslovich TM, Mah AK, Johnsen RC, Cavender JC, Lewis RA, Leroux MR, Beales PL, Katsanis N (2003). Basal body dysfunction is a likely cause of pleiotropic Bardet-Biedl syndrome. Nature.

[104] Badano JL, Leitch CC, Ansley SJ, May-Simera H, Lawson S, Lewis RA, Beales PL, Dietz HC, Fisher S, Katsanis N (2006). Dissection of epistasis in oligogenic Bardet-Biedl syndrome. Nature.

[105] Kim JC, Badano JL, Sibold S, Esmail MA, Hill J, Hoskins BE, Leitch CC, Venner K, Ansley SJ, Ross AJ, Leroux MR, Katsanis N, Beales PL (2004). The Bardet-Biedl protein BBS4 targets cargo to the pericentriolar region and is required for microtubule anchoring and cell cycle progression. Nat. Genet.

[106] Kim JC, Ou YY, Badano JL, Esmail MA, Leitch CC, Fiedrich E, Beales PL, Archibald JM, Katsanis N, Rattner JB, Leroux MR (2005). MKKS/BBS6, a divergent chaperonin-like protein linked to the obesity disorder Bardet-Biedl syndrome, is a novel centrosomal component required for cytokinesis. J. Cell Sci.

[107] Li JB, Gerdes JM, Haycraft CJ, Fan Y, Teslovich TM, May-Simera H, Li H, Blacque OE, Li L, Leitch CC, Lewis RA, Green JS, Parfrey PS, Leroux MR, Davidson WS, Beales PL, Guay-Woodford LM, Yoder BK, Stormo GD, Katsanis N, Dutcher SK (2004). Comparative genomic identification of conserved flagellar and basal body proteins that includes a novel gene for Bardet-Biedl syndrome. Cell.

[108] Jin H, White SR, Shida T, Schulz S, Aguiar M, Gygi SP, Bazan JF, Nachury MV (2010). The conserved Bardet-Biedl syndrome proteins assemble a coat that traffics membrane proteins to cilia. Cell.

[109] Loktev AV, Zhang Q, Beck JS, Searby CC, Scheetz TE, Bazan JF, Slusarski DC, Sheffield VC, Jackson PK, Nachury MV (2008). A BBSome subunit links ciliogenesis, microtubule stability, and acetylation. Dev. Cell.

[110] Nachury MV, Loktev AV, Zhang Q, Westlake CJ, Peränen J, Merdes A, Slusarski DC, Scheller RH, Bazan JF, Sheffield VC, Jackson PK (2007). A core complex of BBS proteins cooperates with the GTPase Rab8 to promote ciliary membrane biogenesis. Cell.

[111] Wiens CJ, Tong Y, Esmail MA, Oh E, Gerdes JM, Wang J, Tempel W, Rattner JB, Katsanis N, Park HW, Leroux MR (2010). Bardet-Biedl syndrome-associated small GTPase ARL6 (BBS3) functions at or near the ciliary gate and modulates Wnt signaling. J. Biol. Chem.

[112] Gerdes JM, Liu Y, Zaghloul NA, Leitch CC, Lawson SS, Kato M, Beachy PA, Beales PL, DeMartino GN, Fisher S, Badano JL, Katsanis N (2007). Disruption of the basal body compromises proteasomal function and perturbs intracellular Wnt response. Nat. Genet.

[113] Corbit KC, Shyer AE, Dowdle WE, Gaulden J, Singla V, Chen MH, Chuang PT, Reiter JF (2008). Kif3a constrains beta-catenin-dependent Wnt signalling through dual ciliary and non-ciliary mechanisms. Nat. Cell Biol.

[114] Ross AJ, May-Simera H, Eichers ER, Kai M, Hill J, Jagger DJ, Leitch CC, Chapple JP, Munro PM, Fisher S, Tan PL, Phillips HM, Leroux MR, Henderson DJ, Murdoch JN, Copp AJ, Eliot MM, Lupski JR, Kemp DT, Dollfus H, Tada M, Katsanis N, Forge A, Beales PL (2005). Disruption of Bardet-Biedl syndrome ciliary proteins perturbs planar cell polarity in vertebrates. Nat. Genet.

[115] May-Simera HL, Kai M, Hernandez V, Osborn DPS, Tada M, Beales PL (2010). Bbs8, together with the planar cell polarity protein Vangl2, is required to establish left-right asymmetry in zebrafish. Dev. Biol.

[116] Kim SK, Shindo A, Park TJ, Oh EC, Ghosh S, Gray RS, Lewis RA, Johnson CA, Attie-Bittach T, Katsanis N, Wallingford JB (2010). Planar cell polarity acts through septins to control collective cell movement and ciliogenesis. Science.

[117] Huang P, Schier AF (2009). Dampened Hedgehog signaling but normal Wnt signaling in zebrafish without cilia. Development.

[118] Ocbina PJ, Tuson M, Anderson KV (2009). Primary cilia are not required for normal canonical Wnt signaling in the mouse embryo. PLoS One.

[119] Wallingford JB, Mitchell B (2011). Strange as it may seem: the many links between Wnt signaling, planar cell polarity, and cilia. Genes Dev.

[120] Bukanov NO, Smith LA, Klinger KW, Ledbetter SR, Ibraghimov-Beskrovnaya O (2006). Long-lasting arrest of murine polycystic kidney disease with CDK inhibitor roscovitine. Nature.

[121] Seeley ES, Carrière C, Goetze T, Longnecker DS, Korc M (2009). Pancreatic cancer and precursor pancreatic intraepithelial neoplasia lesions are devoid of primary cilia. Cancer Res.

[122] Schraml P, Frew IJ, Thoma CR, Boysen G, Struckmann K, Krek W, Moch H (2009). Sporadic clear cell renal cell carcinoma but not the papillary type is characterized by severely reduced frequency of primary cilia. Mod. Pathol.

[123] Chizhikov VV, Davenport J, Zhang Q, Shih EK, Cabello OA, Fuchs JL, Yoder BK, Millen KJ (2007). Cilia proteins control cerebellar morphogenesis by promoting expansion of the granule progenitor pool. J. Neurosci.

[124] Spassky N, Han YG, Aguilar A, Strehl L, Besse L, Laclef C, Ros MR, Garcia-Verdugo JM, Alvarez-Buylla A (2008). Primary cilia are required for cerebellar development and Shh-dependent expansion of progenitor pool. Dev. Biol.

[125] Han YG, Kim HJ, Dlugosz AA, Ellison DW, Gilbertson RJ, Alvarez-Buylla A (2009). Dual and opposing roles of primary cilia in medulloblastoma development. Nat. Med.

[126] Han YG, Alvarez-Buylla A (2010). Role of primary cilia in brain development and cancer. Curr. Opin. Neurobiol.

[127] Gherman A, Davis EE, Katsanis N (2006). The ciliary proteome database: an integrated community resource for the genetic and functional dissection of cilia. Nat. Genet.

[128] Boehlke C, Kotsis F, Patel V, Braeg S, Voelker H, Bredt S, Beyer T, Janusch H, Hamann C, Gödel M, Müller K, Herbst M, Hornung M, Doerken M, Köttgen M, Nitschke R, Igarashi P, Walz G, Kuehn EW (2010). Primary cilia regulate mTORC1 activity and cell size through Lkb1. Nat. Cell Biol.

